# Clinicians’ views and experiences of prescribing oral anticoagulants for stroke prevention in atrial fibrillation: A qualitative meta-synthesis

**DOI:** 10.1371/journal.pone.0232484

**Published:** 2020-05-07

**Authors:** Ruth V. Pritchett, Joanne L. Clarke, Kate Jolly, Danielle Clarkesmith, Danai Bem, Grace M. Turner, G. Neil Thomas, Deirdre A. Lane

**Affiliations:** 1 Institute of Applied Health Research, University of Birmingham, Birmingham, England, United Kingdom; 2 Liverpool Centre for Cardiovascular Science, University of Liverpool and Liverpool Heart and Chest Hospital, Liverpool, England, United Kingdom; 3 Department of Clinical Medicine, Aalborg Thrombosis Research Unit, Aalborg University, Aalborg, Denmark; Inselspital Universitatsspital Bern, SWITZERLAND

## Abstract

**Background:**

Globally, over 33 million people have atrial fibrillation (AF). In eligible patients, oral anticoagulation (OAC) is recommended for stroke risk reduction. Despite recent increases in OAC prescribing, global under-prescription to high-risk AF patients and inappropriate prescription to low-risk patients is leading to unnecessary risk of stroke and haemorrhage. This meta-synthesis explored clinicians’ beliefs and experiences regarding OAC prescription to AF patients, highlighting barriers to stroke prevention and informing future clinician-focused interventions.

**Methods and results:**

A qualitative meta-synthesis exploring clinicians’ views and experiences of prescribing OACs for stroke prevention in AF patients. Databases including MEDLINE, EMBASE, PsychINFO and CINAHL were searched to June 2018, with a further Medline search to February 2020. Thematic synthesis was performed with data coding, descriptive theme categorisation and generation of analytical themes. From 3499 records, 101 full text papers were screened, with 13 eligible studies identified. Four analytical themes were found to affect clinicians’ prescribing: (i) ‘Clinicians’ intellectual and emotional responses to the evidence’; (ii) ‘Prescribing in primary and secondary care’; (iii) ‘Clinicians’ views of how patients’ characteristics and opinions influence prescribing’; and (iv) ‘Clinicians’ views on their interactions with patients’.

**Conclusions:**

This review highlights focal points for future clinician-focused interventions to improve guideline-adherent OAC prescription in AF patients. Interventions should aim to improve clinicians’ knowledge around NOAC prescription and stroke and haemorrhage risk assessment tools as well as their emotional responses to difficult prescribing scenarios. Multidisciplinary interventions promoting cohesive care and input from different clinicians to overcome time-related barriers may increase guideline-adherent OAC prescription for AF patients.

## Introduction

Atrial fibrillation (AF) affects over 33 million people worldwide with prevalence rising globally. [1, 2] Oral anticoagulation (OAC) is recommended for stroke risk reduction by current national and international guidelines for female AF patients with a CHA_2_DS_2_-VASc score ≥2 and male patients with a score ≥1. [3–7] OAC prescription rates are currently around 80% in Europe and the USA, [8, 9] but substantially lower in many low and middle-income countries, including in China (1–4%) and high income Asian countries such as South Korea (32–37%). [10] There are also specific improvements to be made in ‘guideline-adherent’ OAC prescription. Only 68% of the UK’s high-risk AF patients (CHA_2_DS_2_-VASc score ≥2) are receiving OACs, falling to 48% in the Ukraine and 40% in India. [9] Globally, half of all newly diagnosed AF patients with a CHA_2_DS_2_-VASc score of 0 (low risk) are receiving OACs unnecessarily, putting them at increased risk of major haemorrhage. [9]

An understanding of clinicians’ views and experiences of prescribing OAC in practice, including what drives their prescribing decisions and their beliefs regarding barriers to guideline-adherent prescribing, and is necessary to inform the design of future effective interventions supporting clinicians in their management of AF patients.

A 2012 qualitative meta-synthesis explored patients’ and clinicians’ experiences of AF and OACs. [11] Provision of sufficient information; patients’ and clinicians’ preferences for consultation styles; patients’ preconceptions of OACs; and communication between clinicians all affected clinicians’ and patients’ experience of AF and OACs. [11] In 2017, a qualitative meta-synthesis explored AF patients’ and clinicians’ views specifically of vitamin K antagonist (VKA) oral anticoagulants. [12] Authors reported that available scientific information; concerns about risks related to VKAs; confidence in prescribing; and degree of paternalism were concerns pertinent to clinicians and patients’ decision-making processes.[12] In addition, patients placed importance on their own knowledge and understanding of OACs, the impact of OAC on their daily life and satisfaction with their OAC therapy. Despite the continued popularity of VKAs (predominantly warfarin), there has been a considerable rise in NOAC (Non-VKA oral anticoagulants) use, a class of drugs with a different risk profile and very different daily management requirements. This change in prescribing habits resulted in the 2017 [12] review presenting an incomplete picture of the current challenges of OAC decision-making and prescription. To explore potential reasons behind the current disparity between clinicians’ prescription of OACs for AF patients and guideline recommendations, an up-to-date review is required including all currently licenced OACs for stroke prevention in AF.

## Aim of this review

To identify and synthesise qualitative research concerning the views and experiences of clinicians in prescribing OACs for stroke prevention in AF patients. Our findings will highlight barriers to stroke prevention that may help to inform future interventions supporting clinicians in managing AF patients.

## Methods

### Protocol and registration

This systematic review has followed the reporting guidelines formulated in the ‘Enhancing transparency in reporting the synthesis of qualitative research’ (ENTREQ) statement. [13] The protocol was registered with PROSPERO prior to conducting literature searches (CRD42016038133).

### Eligibility criteria

Qualitative studies, standalone or within mixed methods designs, investigating the views and experiences of clinicians in the decision-making process concerning OAC prescription for stroke prevention in AF patients. We have defined ‘clinicians’ as any healthcare professional licenced to prescribe OACs to AF patients.

### Literature searches

A comprehensive, pre-planned search strategy employed search filters and/or index terms relating to "qualitative research". A combination of text words and index terms were used relating to “atrial fibrillation", “oral anticoagulants” and "views, experiences, perceptions and attitudes" (see S1 Fig). No language or date restrictions were applied to searches which included all material published from the inception of each database to 12^th^ June 2018. MEDLINE, MEDLINE in Process, EMBASE, PsychINFO, CINAHL, Applied Social Sciences Index and Abstracts (ASSIA) were searched for primary studies; Sciences and Social Sciences Citation Index (Web of Science) for citation searching. Grey literature sources were Open Grey, Sociological abstracts, conference proceeding citation index (Web of Science) and the Healthcare Management Information Consortium (HMIC). Citation lists of included studies and relevant reviews were checked. An update of the main Medline search was conducted from inception to 3^rd^ February 2020.

### Study selection process

**Fig 1:**
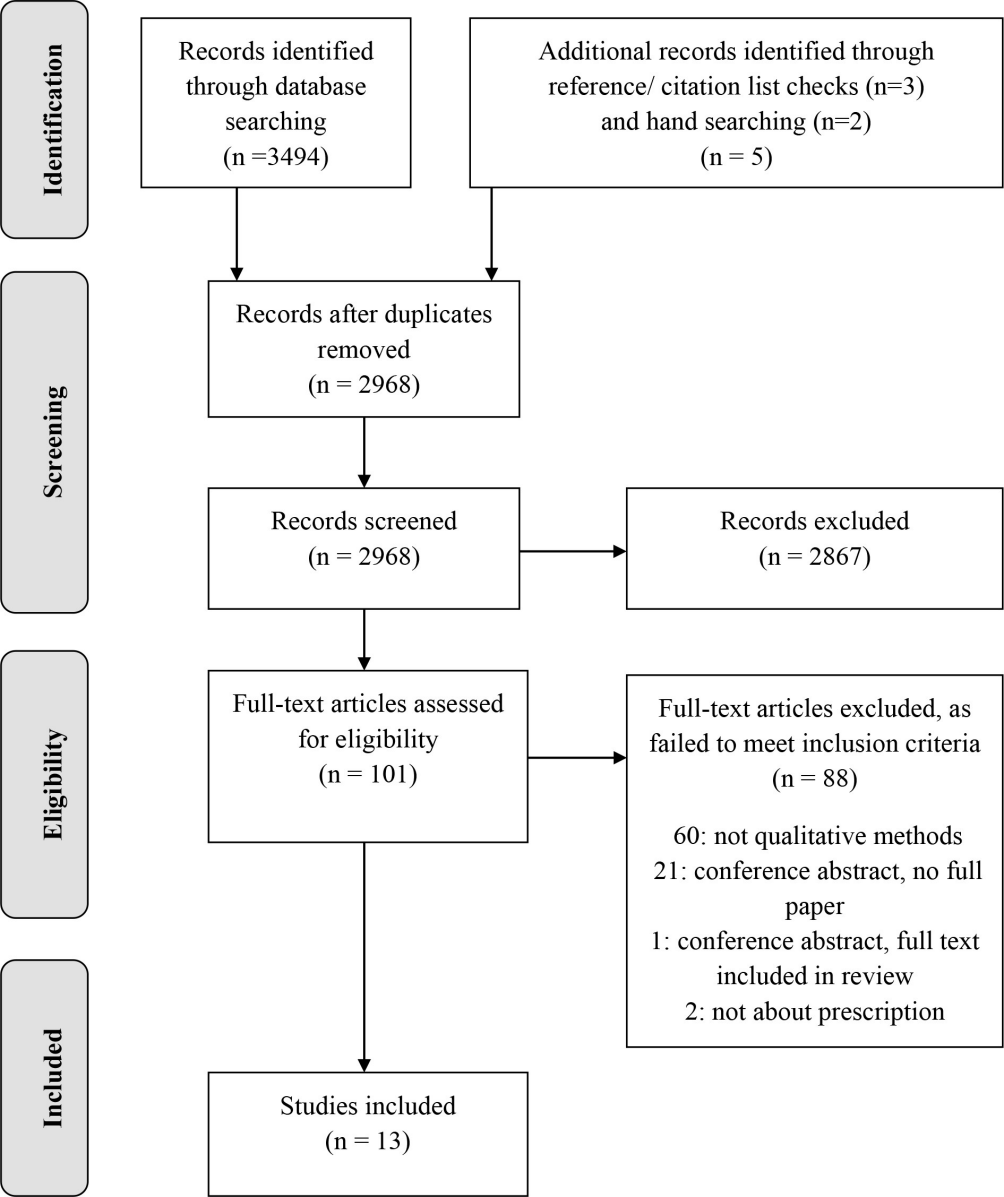
PRISMA flow chart for study selection.

Search results were exported into Endnote X8.0.2 and duplicates removed. Two reviewers (JC and DC/RP) independently screened titles and abstracts and assessed full texts of potentially relevant studies against inclusion criteria. Disagreements were discussed with a third reviewer (GT). Authors of relevant conference abstracts were contacted to see if work had been published, with requests for access to unpublished data, where appropriate. The study selection process is illustrated in a PRISMA flow diagram (Fig 1).

### Data extraction

Data from included studies were extracted independently by two reviewers (JC and RP) using a standardised piloted data extraction form containing details regarding the study’s year of publication; aim; where it was conducted and in what type of setting; participant demographic characteristics; the qualitative method used; the data analysis method and the findings. Discrepancies were resolved through discussion and comparison with original data. Authors were contacted for clarification as required. The following information was extracted from all included studies: study characteristics and location, aim; participants (e.g. number, recruitment/sampling strategies); qualitative approach, research paradigm, (e.g. grounded theory, ethnography, phenomenology) and methods (e.g. survey); data analysis methods and findings (including quotes and authors’ interpretations).

### Quality assessment

Two reviewers (JC and RP) independently assessed the methodological quality of included studies using the Critical Appraisal Skills Programme (CASP) Qualitative Research Checklist. [14] Disagreements were resolved by discussion. Studies were not excluded based on quality or adequacy of reporting.

### Data synthesis

An inductive three-stage thematic synthesis of qualitative data was undertaken. [15] First, line-by-line coding of the findings of primary studies was conducted, according to meaning and content. All findings, in both quote form and author comments, were coded, as recommended by Thomas and Harden. [15] Coding was conducted independently by two researchers (RP and JC) using NVivo version 12; [16] interpretations were compared and consensus reached through discussion. Translation of concepts between studies was achieved by applying the codes developed through coding initial transcripts, to subsequent transcripts, with new codes being created where necessary. Codes were then categorised into descriptive themes. Finally, analytical themes were developed to describe and understand all previous descriptive themes.

## Results

This review identified 3499 records. After removing duplicates, a further 2867 ineligible records were excluded. The eligibility of 101 full text papers was assessed, with 13 studies being included (Fig 1). For details of excluded full text papers see S1 Table in S1 File.

### Study quality

Included studies varied in rigour according to the CASP qualitative checklist. [14] Ten studies were high quality in relation to the majority (five or more) of the nine graded fields of the qualitative CASP checklist. [17–26] Poorly described facets included the relationship between researcher and participant; [17–23, 25–29] explanation of the study to participants and handling of their data; [17, 19–21, 23–25, 27–29] rigour of data analysis [18–23, 25, 27–29] and appropriateness of recruitment strategies. [19, 21, 22, 25, 27–29] For further details of study quality see S2 Table in S1 File.

### Study characteristics

Characteristics of included studies are reported in detail in S3 Table in S1 File and summarised in Table 1. Data were included from at least 248 clinicians. Included studies were conducted in the UK, [19, 21, 22, 24] Finland, [29] Australia, [17, 20, 27] the USA [18, 23, 25, 26] and Canada. [28] Participating clinicians were from primary, [17, 19, 20, 22–25, 28, 29] secondary [18–21, 23, 25, 26, 28, 29] and tertiary care. [18, 21, 25, 27] Qualitative methodologies included focus groups, [22] semi-structured interviews, [19, 20, 24–26, 29] in-depth interviews with a semi-structured guide [18] and interviews and reviews of healthcare file notes. [27] Mixed-methods studies included semi-structured interviews exploring decision-making in relation to vignettes, [21] surveys and interviews [23, 28] and questionnaires with both qualitative and quantitative responses. [17]

**Table 1:** Characteristics of included studies.

Study characteristics	Participants	Qualitative method
Freeman et al. 2001 [22]	Primary care	A series of focus groups with case note discussion.
	19 GPs	
South West England, UK		Facilitated by a group leader GP
Lipman et al. 2004 [24]	Primary care	Semi-structured interviews conducted by a GP
	12 GPs (1 poor recording quality interview discarded)	
Northern England, UK		
Anderson et al. 2007 [21]	Secondary and tertiary care	Semi structured interviews considering treatment decision related to vignettes.
	14 senior consultants/specialist registrars: 5 in Cardiology; 9 in general medicine/geriatric medicine.	
Leeds, UK		
		Conductor of interviews unclear.
Murray et al. 2011 [28]	Primary and secondary care	Open-ended, semi-structured telephone interviews.
	28 clinicians interviewed: family physicians/GPs; internal medicine specialists; cardiologists; emergency physicians and neurologists.	
Sites across Canada		Conductor of interviews unclear.
Decker et al. 2012 [18]	Secondary and tertiary care	In-depth interviews in person or via telephone conducted by the study coordinator.
A large Midwestern city, USA	27 clinicians: 18 cardiology physicians; 3 cardiology nurses; 5 internal medicine physicians; 1 internal medicine nurse practitioner.	
		Semi-structured interview guide used.
Bajorek et al. 2015 [17]	Primary care	A structured questionnaire with both quantitative and qualitative responses, on paper, in person.
	50 GPs from ‘General practice’ or ‘Medical locals’.	
New South Wales, Australia		
Borg-Xuereb et al. 2016 [19]	Primary and secondary care	Semi-structured interviews.
	16 clinicians: 4 consultant cardiologist; 4 consultant general physicians; 4 general practitioners; 4 cardiology registrars.	Conductor of interviews unclear.
West Midlands, UK		
Kirley et al. 2016 [25]	Primary, secondary and tertiary care	Semi-structured open-ended interviews conducted by the primary author.
USA	7 clinicians: 3 family physicians; 1 internist; 2 cardiologists; 1 cardiologist specialising in electrophysiology.	
Wang et al. 2016 [20]	Primary and secondary care	Semi-structured face-to-face interviews with open-ended questions were conducted by a researcher.
Sydney, Australia	26 clinicians: Seven pharmacists; seven specialist clinicians; 6 GPs and six nurses.	
Karcher et al. 2016 [23]	Primary and secondary care	One-to-one interviews using standardised questionnaires including props to facilitate expansion if needed
	14 clinicians interviewed: 7 physicians (from cardiology or primary care practices), 5 nurses, 2 medical assistants.	
11 states in South Eastern USA		
		Conductor of interviews unclear.
Ferguson et al. 2017 [27]	Tertiary care	Patient interviews during bed-side clinical assessments and review of healthcare file notes.
	Clinician’s notes from the treatment of 144 patients with Chronic heart failure and concomitant AF.	
Sydney, Australia		
		Conductor of interviews unclear.
Aarnio et al. 2019 [29]	Primary and secondary care	Semi-structured interviews with open-ended questions. 13 face-to-face and 4 phone interviews conducted by 1 researcher.
Central and Eastern Finland	17 HCPs: 8 GPs; 2 neurologists; 5 cardiologists; 2 internal medicine specialists.	
Kea et al. 2019 [26]	Secondary care	Semi-structured telephone interviews conducted by a resident physician skilled in conducting interviews.
Oregon, USA	18 HCPs: Emergency department attending physicians	

### Summary of themes

**Fig 2:**
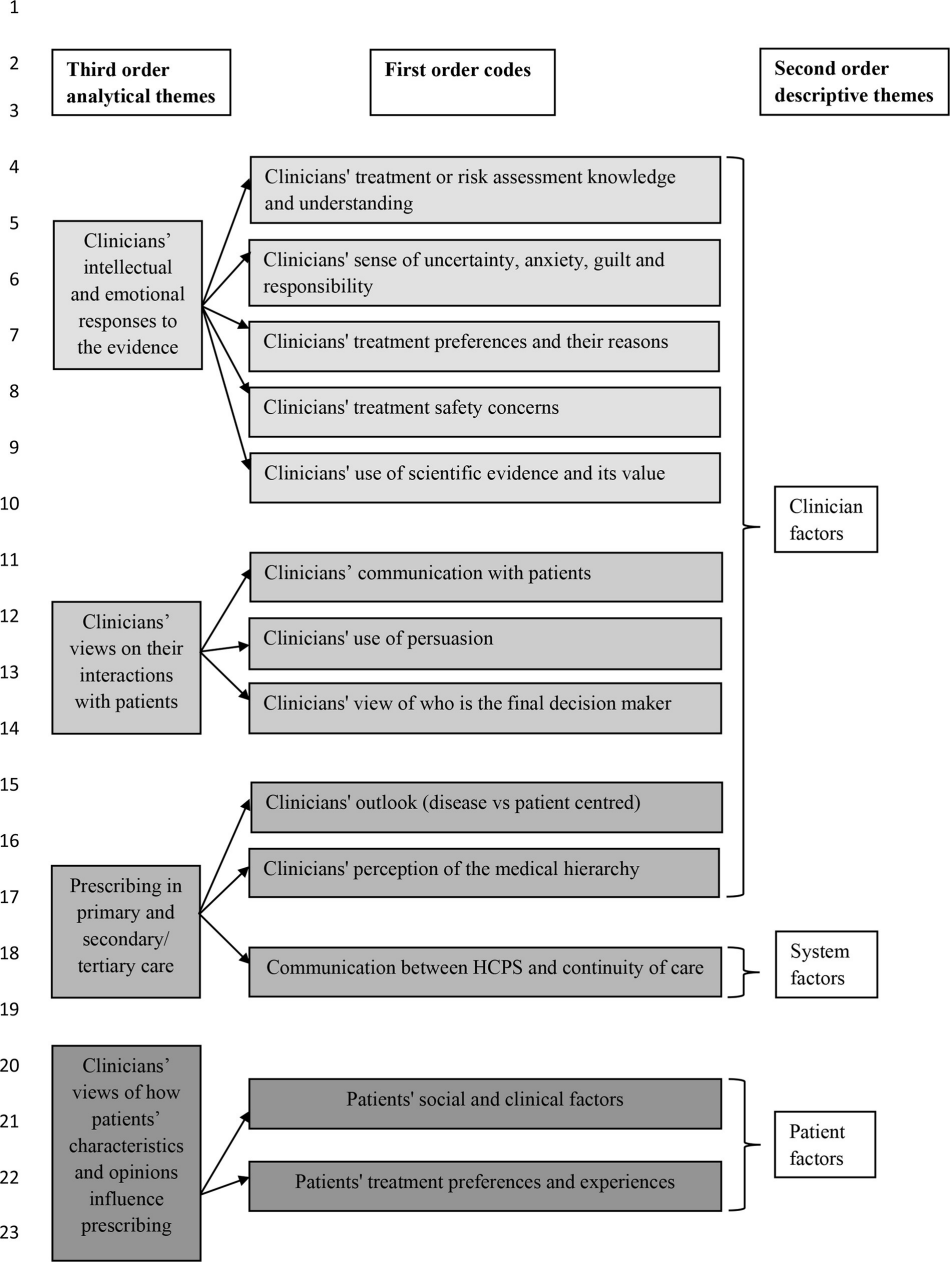
Codes, descriptive and analytical themes.

Four analytical themes emerged from the data: (i) ‘Clinicians’ intellectual and emotional responses to the evidence’; (ii) ‘Prescribing in primary and secondary/tertiary care’ (iii) ‘Clinicians’ views of how patients’ characteristics and opinions influence prescribing’; and (iv) ‘Clinicians’ views on their interactions with patients’. (For summary of codes, descriptive and analytical themes see Fig 2; for frequency of codes within studies see S4 Table in S1 File)

## Qualitative synthesis

### Clinicians’ intellectual and emotional response to the evidence

Clinicians’ interpretations of the evidence guiding the change in stroke prevention over the past decade from anti-platelets and VKAs to VKAs and NOACs varied greatly and were influenced by the evidence and the opinions of hospital physicians and patients. [24, 26] Clinicians justified their differing views with selective use of supportive evidence. Clinicians’ uncertainty in prescribing OAC also resulted from a perceived lack of evidence to guide choices between warfarin and the various NOACs.

*“The other pro-warfarin health professionals were either uncertain about the efficacy and bleeding risks of NOACs compared with the risks associated with warfarin*, *or followed specific guidelines and information from continuing medical education that favoured warfarin*.*”* [20]

The accessibility of the format and content of the guidelines were raised. Some clinicians made a case for creating guidelines specific to different disciplines.

*“there should be one A4 page for an emergency department medical assessment unit*, *a different A4 for cardiology clinics*, *different A4 for general practice*, *and different one for general physicians*. *All probably saying the same thing*, *but the way it is being put across*, *the simplicity is the important point*. *[D9*, *General physician]”* [19]

In addition to their considered intellectual response to scientific evidence, some clinicians described their emotional response to the evidence.

*“There was some ambiguity in the approach to guidelines*. *Three respondents were broadly in favour of guidelines (GP5…)*, *one applied them ‘flexibly’ and three were largely hostile*, *(GP2)*.*”* [24]

Some clinicians reported discomfort with the rigid nature of guideline criteria. Others reported anxiety, both within themselves and from their patients, when they felt that the evidence called for a change of practice.

*“Yes it does make me feel anxious…all the BMJs*, *all the rags…these people must be on warfarin…With me messing about with his medication and trying to practise evidence based medicine*, *I found it was making him [patient] feel more anxious*.*”* [22]

Clinicians also reported feeling anxious about potentially causing harm to patients and being held accountable for such harm.

*“I think doctors are anxious about avoiding risks of medications*, *we worried about causing bleeding*. *But because of our fear of that we massive under treat patients with AF and we don’t prevent stroke when we should be…because our failing as a medical profession is about not using these drugs enough…I think the disaster is that the patient has a stroke because they had an untreated AF*. *(G01)”* [20]

Sometimes safety concerns, such as those regarding falls and haemorrhage, led to inappropriate prescribing, however other concerns did not.

*“‘I almost feel like it’s the reverse*, *where the more I know*, *the more fear I have of using it*. *The sense of first do no harm*. *(IM physician*, *25)’ ‘Coumadin is a terrible drug to take*, *but it’s the least of two evils*. *So we have to do it*. *(Cardiologist*, *10)’”* [18]

For some this anxiety was based on previous negative experiences with OACs. Guilt due to personal experiences was a strong motivator.

*“I actually had two 50 year olds who had strokes from atrial fibrillation because they didn’t get warfarin*… *that really hit me*.*”* [22]

The degree to which stroke and haemorrhage risks were assessed and influenced the prescribing process varied substantially. Stroke risk was often a more important consideration than bleeding risk. The use of guideline-based risk assessment tools such as the CHA_2_DS_2_-VASc and HAS-BLED was not common practice for all clinicians.

*“most PCP [primary care physicians] interviewees were unfamiliar with both the CHA*_*2*_*DS*_*2*_*-VASc score and bleeding risk assessment tools”* [23]

Some clinicians reported a lack of knowledge of stroke and haemorrhage risks in specific cases and the skills required to individualise treatment.

*“If he had a metallic valve*…*I don't think I'd be comfortable to make that decision*. *I don't know what the risk of having a stroke is*…*I wouldn't discount home being on warfarin*, *but only because it's a kind of imprint”* [21]

Clinicians’ described different treatment preferences, sometimes based on clinical considerations, sometimes formed through habit.

*“it goes back to the culture thing*, *if you have cardiologist who have been using warfarin for the last 40 years and why they are going to change to NOACs*?*”* [20]

Regular warfarin monitoring was sometimes seen as providing a safety net unavailable to patients on NOACs; however, the risk of bleeding and poor anticoagulation control (international normalised ratio, INR) associated with VKAs were off-putting to some clinicians. Some clinicians expressed concern about lack of long-term safety data for NOACs, particularly in older patients, and their lack of reversibility; however, others reported a preference for NOACs, due to their more stable INR, the lack of regular testing and their superior effectiveness and safety compared to warfarin. Often, no general consensus was reached regarding the choice between warfarin and the various NOACs.

*“11 physicians considered high-quality warfarin treatment as effective as DOACs*, *although 11 physicians felt DOACs to be safer than warfarin*. *In addition 10 physicians mentioned that they did not see great differences between different DOACs or that they have not been directly compared and*, *therefore*, *none of them can be said to be better than the others’* [29]

Different types of clinicians described different opinions of NOACs and warfarin.

“*compared with cardiologists and geriatricians*, *who were cautious about using NOACs*, *a neurologist and the haematologists had strong opinions about using NOACs as first-line therapy*.” [20]

A perception was reported that GPs were quite reluctant to use warfarin.

*“even before the NOACs patients still were not put on to warfarin who had AF*, *just because they (GPs) thought the risk of bleeding is far too high*.*”* [20]

### Prescribing in primary and secondary/tertiary care

A well-developed doctor-patient relationship and the perception of less time-pressured consultations in primary care were thought to enable more thorough treatment discussions, however, specialist knowledge was seen to be lacking compared to secondary care. In contrast, lack of time in secondary care was seen to seriously affect patient education and involvement in decision-making.

*“In our chronic disease clinics … the people with AF on warfarin will see the same practitioner … it’s a very good system … helps build a very good relationship […] I know how they live*, *where they live*, *their family members*, *what support system they have got*. *As well as the medical history I know their social history*. *[D16*, *GP]”* [19]

*“Patient education is very important*, *but it is one that we do badly*, *because there isn’t enough time*. *In general I see about 15 to 16 patients in 3 h … so there is not enough time to talk about AF*, *talk about what you’re gonna do …and then educate patients as well*. *[D8*, *Cardiology registrar]”* [19]

Perceived strengths and weaknesses of different healthcare settings were seen to be reflected in the different outlooks of the clinicians.

*“hospital doctors tended to impose a decision ‘treating the disease rather than the patient*.*’… the idea that there is a treatment of a condition therefore you must treat patients with this condition with this treatment is what a lot of hospital doctors think …whereas I think in general practice people often are prepared to take a step back and say ‘Well yes*, *there is a treatment there but is it in the best interests of this patient*, *is it going to fit in with the rest of you know*…*it’s the holistic view’” (GP11)* [24]

The availability of medical care was also seen to influence the prescribing process.

*“Complications always tended to happen ‘over the weekend*,*’ and those practitioners who*, *for example*, *did not always have nursing staff to help do blood tests seemed to be less enthusiastic about implementing evidence on anticoagulation”* [22]

A lack of effective communication between primary and secondary care was reported.

*“One of the biggest problems we have is a lack of communication*, *too many chefs in the kitchen…it’s a waste of my time to do all the research and then send patients to somebody who feels uncomfortable with my plan”*[26]

Clinicians sometimes reported confusion over where the principal responsibility for OAC prescribing lay, with some clinicians routinely referring their patients for anticoagulation, others for support of their recommendations and some referring only complex cases. This disjointed care pathway was seen to have a serious impact on accountability.

*“Decision making for who goes on warfarin is taken often by one person*, *monitoring of warfarin is taken by another person and in our practice people are monitored in 5 different systems…ongoing responsibility for patient education is non-existent…the potential risks of warfarin to me are so large in terms of errors basically*. *(GP2)”* [24]

GPs reported feeling uncomfortable challenging the decisions of consultants, who they regarded as experts. GPs saw this conflict as being an issue for themselves and their patients, who they felt were more inclined to take their consultants advice.

*“the one thing that surprised me looking through this case note review is just how big an influence secondary care is still having on the decision as to what people have*, *whether they get anticoagulated or not and how difficult it is in practice to change that*.*” (GP5)* [24]

However, this view of the medical hierarchy was sometimes felt to be mediated by the individual confidence of the clinician with OAC prescription. Amongst emergency department clinicians a relationship was found between overall lack of experience and deference to cardiologists in OAC prescription.

*“Physicians with less than 10 years of experience more commonly indicated that they would consult a cardiologist to gain concordance of opinion”* [26]

### Clinicians’ views of how patient characteristics and opinions influence prescribing

Clinicians’ views of how medical factors such as patients’ age, mental state, fall risk, other medications and medication adherence influenced prescribing, were widely discussed. Clinicians also acknowledged the burden of INR testing and questioned patients’ drive, cognitive ability, mental health, and access to the social and medical structure needed to successfully take OACs. Great emphasis was given to the psychosocial suitability of patients by clinicians.

*“I add up their CHADS*_*2*_ *score and think about what category they fall into*. *I also am concerned with the social milieu of the patient–do they have the will*, *the intelligence*, *how far are they from medical care*. *Those things factor very strongly into my decisions (IM physician*, *25)”* [18]

Equally, patient refusal often highlighted the importance of non-clinical factors to patients. However, this was reportedly not always taken into account within the consultation discourse.

*“Patient stories about refusing warfarin demonstrate that lifestyle needs and choices were significant to their decision*. *Yet physicians’ information provision focused on explaining the physiology of the condition and the stroke risk reduction associated with warfarin*.*”* [19]

Clinicians sometimes reported conflict between the guidelines and the influence of patients’ psychosocial factors. On occasion, a clinician’s response to patient’s psychosocial factors resulted in guidelines potentially not being followed.

*“‘Strictly speaking this chap could get by without any anticoagulation but in view of his anxiety*…*I think I would go ahead and offer him long term warfarinization*.*”* [21]

Some American clinicians described perceiving racial or socioeconomic prejudice among colleagues. It was reported that some clinicians’ opinions regarding non-compliance in certain patient populations were not supported with evidence.

*“None of the interviewees reported personal racial biases; yet when asked for reasons why African Americans may have a higher incidence of AF-related stroke and related mortality*, *many responded that it was because of a higher rate of noncompliance in this patient population*. *However*, *no specific examples or data supporting this view were provided*.*”* [23]

For some clinicians their assumptions about patients’ lack of understanding about OAC treatment influenced their willingness to prescribe OAC and undertake the required monitoring.

*“you have listed all possible risk factors (stroke*, *bleeding and medication safety risk factors)…So it is just you would have to constantly measure these from time to time*. *It is time consuming and detailed and maybe doesn’t warrant maybe because of the patient health illiteracy*. *(P05)”* [20]

Physicians from countries in which patients were directly or indirectly (through insurance) charged for OAC medication reported that the cost of NOACs could form a prohibitive barrier that outweighed both clinical and social considerations.

*“Some patients can’t really afford NOACs*. *I think they’re something like 90 bucks a month or something*. *So I wouldn’t switch anyone over basically because of the cost difference*.*”* [25]

Patient beliefs could also influence the prescribing process. Clinicians noted a dislike of warfarin amongst patients due to its use as a rat poison and the burden of frequent blood test monitoring, prompting a preference for NOACs.

*“Taking something like warfarin is incredibly taxing on people…with weekly or at least biweekly INR checks’* [26]

### Clinicians’ views on their interactions with patients

Clinicians employed a wide range of methods to convey the physiology of patients’ stroke and haemorrhage risks including risk and benefit language, metaphors, description of physiology, drawings and printed information. However, providing explanations of AF and the associated risks to asymptomatic patients was challenging for some. There was a wide range in the degree of information clinicians felt their patients required.

*“two GPs described minimal explanation of risk and benefit to patients…five gave some detail and three described detailed and explicit explanation”* [24]

*“The only way you can empower the patient to make decisions is to provide the information that they require to make that decision*.*”* [19]

Discussion of the motivation behind clinicians’ interactions with patients revealed a desire to persuade patients of the ‘right’ decision.

*“Some GPs described quite strong levels of persuasion while others were content to explain their view of the risks and benefits and assist the patient to choose*. *Equally*, *while some GPs described negotiating the decision*, *others seemed to be describing exerting pressure on patients to accept responsibility for the decision or*, *conversely*, *to agree to the choice the GP thought most beneficial*.*”* [24]

Persuading the patient to make ‘the right treatment decision’ sometimes involved careful choice of language.

*“The choice of words or the use of metaphors like ‘slanting’ or ‘selling’ were mechanisms the doctors used to influence patients to make a decision about their treatment that was consistent with what the doctor had decided was appropriate*. *Doctors would refer to ‘rat poison’ when describing warfarin if they felt its use would be difficult or inappropriate*, *or describe pills as ‘having been shown to keep the heart young’ when they wanted a patient to agree to treatment*.*”* [22]

Selective provision of information either, to avoid provoking fear, or capitalise on it in order to encourage patients to make ‘the right treatment choice’ was described.

*“I don’t usually warn them about brain haemorrhage … perhaps I should*. *But I don’t*. *[D3*, *Consultant cardiologist]”* [19]

*“I mean generally*, *if you tell them it’s a stroke*, *they get worried about it and generally as you know*, *when people think of stroke*, *they think ‘oh my god*, *I’ll become bed bound*, *very disabled*, *I wouldn’t be able to do it’*. *So they start to take it very seriously that AF is not as benign as they expected*. *[D10*, *General physician]”* [19]

Clinicians were very aware of the concepts of patient-centred and shared decision-making. Consultation styles varied from strongly patient-centred to strongly paternalistic.

*“GPs influencing patients by a combination of persuasion*, *shared decision making*, *GP- or patient-centred consulting*, *and explanation*, *with the GPs’ input being modified by their view of the evidence*. *The weight and quality of each component varied from one GP to another (and possibly from one consultation to another)*.*”* [24]

Some clinicians were comfortable allowing patients’ views and experiences to guide the treatment decision.

*“I think it’s a personal decision rather than a right decision…their life and how they feel about life*, *how they feel about death*, *how they feel about illness and all those sort of things to actually throw that into the equation” (GP8)* [24]

Clinicians were more able to accept a patient’s decision to decline treatment if they believed the decision was well informed.

*“GPs with most experience of EBM were also most willing to engage with the agendas of patient-centred consulting and shared decision making*, *and were most relaxed if patients declined treatment*, *provided they were convinced that the patient understood the consequences…they were willing to question the applicability of guidelines to individual patients and believed that patients would sometimes accept higher levels of risk than suggested by guideline treatment thresholds”* [24]

For some clinicians, the acceptability of the treatment options to the patient was important, with the final decision being shared. Some clinicians found that patient involvement in the decision making process had beneficial long-term effects.

“three physicians felt that patients adhere better to their treatment when they have participated in the decision-making” [29]

Some clinicians began discussing their beliefs regarding the importance of a patient-centred approach; however, their thoughts then led on to the importance of making the right decision and revealed a much more paternalistic style that many patients were reportedly happy with.

*“The only way you can empower the patient to make decisions is to provide the information that they require to make that decision*. *AND if you don’t then they make the decision on the basis on inaccurate or inadequate information and they often come to the wrong conclusion…but erm the majority just allows me to choose what’s best for them*. *[D1*, *Consultant cardiologist]”* [19]

Other clinicians were confident in their opinion and presented the options as a fait accompli.

*“My own personal view I would probably avoid warfarin lifelong so the option would either be aspirin or clopidogrel*.*”* [21]

Some clinicians who were certain in their belief in the right treatment decision, apportioned blame to themselves if their advice was not followed and experienced a sense of failure.

*“I’ve made a mistake in how I’ve described the risk for that individual patient because they made a decision which I think is probably the wrong decision (GP4)”* [24]

## Discussion

This review explored the views and experiences of clinicians prescribing OACs for stroke prevention in AF patients, revealing the barriers to guideline-adherent OAC prescription from the clinician’s viewpoint. Clinicians reported challenges around OAC prescription relating to both their intellectual and emotional response to evidence; the primary and secondary/tertiary care settings; the characteristics and opinions of their patients; the cost of NOACs and their interactions with patients during the prescription consultation.

A wide variation in familiarity with risk assessment tools and greater emphasis on stroke risk rather than bleeding risk assessment was evident. A lack of confidence and knowledge was reported in primary care and amongst emergency department clinicians, especially of risk factors in more complex cases and some clinicians were unsure about the relative merits of warfarin and the various NOACs in different clinical scenarios. Further education and training in using appropriate risk assessment tools may be beneficial to clinicians lacking confidence in their application. Guidelines specific to different healthcare settings/types of clinicians may be welcomed. Our findings support a recent quantitative review of interventions to improve OAC prescription for AF patients, which found educational interventions and the implementation of local guidelines may be effective approaches to improving OAC prescription. [30] A reluctance to prescribe NOACs was also found due to concerns regarding a lack of long-term safety data. Further research into the safety profile of NOACs may improve clinicians’ confidence with these medications.

Clinicians’ treatment preferences were sometimes based predominantly on scientific evidence, such as guidelines, risk assessment scales and knowledge of specific drug risk profiles. Sometimes preferences were based on more subjective considerations, such as perceptions of a patient’s likely adherence to monitoring and treatment. Assumptions about patients’ adherence to medication based on their socioeconomic or ethnic background influenced prescribing in American research. [23] Sometimes treatment preferences were heavily influenced by clinician’s emotional response to treatment decisions, such as fear of causing harm and being held accountable; clinician and patient anxiety at the prospect of new evidence requiring a change in practice; or nervousness about the loss of regular monitoring when moving from warfarin to NOACs. Encouraging greater use of the stroke and haemorrhage risk assessment tools may help to rationalise the prescription process; however, interventions enabling clinicians to explore and challenge their emotional responses to difficult OAC prescribing scenarios may also prove useful.

A perception was reported that primary care fostered a greater understanding of a patients’ social context and more thorough treatment discussions, but provided less specialist knowledge than in secondary care. Secondary care clinicians were reportedly more inclined to treat the ‘condition’. It is not for this paper to discuss the relative merits of the holistic and more condition-focused outlooks, however, an emphasis on patients’ ability to adhere to regular monitoring and maintain a suitable INR may have made some clinicians more cautious in prescribing OACs. Again, encouragement to use suitable risk assessment tools may help to focus prescribing decisions on the evidence. Local interventions to upskill primary care physicians have reported some success in improving rates of appropriate OAC use among AF patients. [31]

Clinicians’ methods and degree of communication with patients regarding AF itself and OAC treatment varied greatly; some clinicians described seeing patients first prescribed OACs in secondary care who were not well informed on their condition and treatment. Time has been reported as a limiting factor in a previous review. [11] Other clinicians, such as pharmacists, may be well placed to provide further, detailed explanations on OAC therapy or to reinforce key messages over time, to encourage adherence. As in previous research, [12] lack of good communication was noted between primary and secondary care. Poor communication was compounded by lack of agreement on treatment; conflict due to the deference of primary care, other non-cardiologist clinicians and their patients to secondary care expertise and a lack of accountability due to the number of people involved in each patient’s care. However, a previous review, [12] despite describing poor communication between primary and secondary care, reported that it was primary care physicians who felt specialists were delegating the responsibility of decision making regarding OAC prescription to them. [12] Greater cohesion of services may be encouraged through interventions that are delivered to interdisciplinary teams from primary, secondary and tertiary care. This finding is supported by current AF guidelines [4, 5] and a recent quantitative systematic review that suggested interdisciplinary medical care programmes educating both clinicians and AF patients were effective in improving OAC prescription. [30]

### Strengths and limitations

This qualitative meta-synthesis was rigorously conducted in accordance with the ENTREQ statement [13]. Searches were free from language bias and duplicate screening, data extraction, quality assessment and coding were used. Included studies were generally of good quality but with poor descriptions of relationships between the researcher and participants, recruitment processes and rigorousness of analyses. The lack of detailed descriptions of these facets of the qualitative research process should temper the weight given to the conclusions presented. All included studies were conducted in high-income countries; therefore, the findings may not be applicable to low and middle-income countries. Included studies were published between 2001 and 2017; changes in the treatment of AF with OACs during this time should be borne in mind when considering the review findings.

## Conclusions

Our findings indicate that despite initial evidence of the benefit of NOACs in older adults (aged ≥75 years), further research into the long-term safety of NOACs amongst older adults would improve some clinicians’ confidence in prescribing them. Our findings also highlight potential areas of focus for future interventions to improve clinicians’ guideline-adherent prescription of OACs for stroke prevention in AF patients. Such interventions should be multi-disciplinary and promote cohesive working due to the range of clinicians involved in managing AF. Interventions should incorporate primary and secondary care physicians, nurses, pharmacists and anticoagulation clinic staff, to help address time limitations of secondary care physicians. Interventions which address clinicians’ knowledge around NOAC prescription and risk assessment tool use; as well as their emotional responses to difficult prescribing scenarios may be important in improving guideline-adherent OAC prescription for stroke prevention in AF patients.

## Supporting information

S1 Checklist PRISMA 2009 checklist. (DOC)

S1 Fig Search strategy. (DOCX)

S1 File (DOCX)
