# Supplementary material for: Clinicians’ views and experiences of prescribing oral anticoagulants for stroke prevention in atrial fibrillation: A qualitative meta-synthesis
Source: PLoS One. 2020 May 7;15(5):e0232484. doi: 10.1371/journal.pone.0232484 (PMC7205240; doi:10.1371/journal.pone.0232484)
Supplement: S1 Fig — (DOCX) [file pone.0232484.s002.docx]

**S1 Fig: Search Strategy**

Ovid MEDLINE(R) <1946 to July Week 1 2016>

1 Atrial Fibrillation/ or "atrial fibrillation".mp. (53050)

2 Atrial Flutter/ or "atrial flutter".mp. (6975)

3 ("auricular fibrillation" or "heart fibrillation" or "heart atrium fibrillation").ti,ab. (870)

4 1 or 2 or 3 (56225)

5 exp Anticoagulants/tu, th [Therapeutic Use, Therapy] (55536)

6 ((anticoagula$ or antithrombotic$1) adj2 (therapy or treatment or under-treatment or uptake or underus$ or prescri$ or prophylaxis or manage$ or assess$ or improve$)).ti,ab. (21939)

7 exp Factor Xa Inhibitors/tu [Therapeutic Use] (315)

8 exp Antithrombins/tu [Therapeutic Use] (2127)

9 ("direct thrombin inhibitor$1" or DTI$1 or "factor Xa inhibitor$1" or "fxa inhibitor$1" or NOAC$1 or "novel oral anticoagulant$1" or "new oral anticoagulant$1" or "non-vitamin K antagonist$1").ti,ab. (11139)

10 exp Coumarins/tu, th [Therapeutic Use, Therapy] (11575)

11 ("Vitamin K antagonist" or VKA$1).mp. (1545)

12 Warfarin/ or (warfarin or C?umadin or Jantoven or Marevan).ti,ab. (22881)

13 ("indandione derivative$1" or "non-c?umarin VKA$1" or 4-hydroxycoumarin$1 or dabigatran or rivaroxaban or apixaban or edoxaban or dic?umarol or phenprocoumon or acenocoumarol or tioclomarol or "ethyl biscoumacetate" or phenindione or clorindion or diphenadion or fluindion$).ti,ab. (6607)

14 5 or 6 or 7 or 8 or 9 or 10 or 11 or 12 or 13 (93267)

15 (perception$1 or experience$1 or perspective$1 or insight$1 or view$1 or opinion$1 or thought$1 or expectation$1 or belie$ or anticipation$1 or attitude$1 or prospect$1 or presumption$1 or observation$1 or lesson$1 or interaction$1 or concept$ or aware$ or feeling$1 or understand$ or knowledge or recogni$ or fear$1 or reluctan$ or hesita$ or position$1 or barrier$1 or facilitator$1 or preference$).mp. (5269831)

16 qualitative research.mp. or exp Qualitative Research/ (33005)

17 focus group$1.mp. or exp Focus Groups/ (29227)

18 nursing methodology research.mp. or exp Nursing Methodology Research/ (16066)

19 (qualitative or "grounded theory" or ethnogra$ or ethnolog$ or phenomenogra$ or phenomenolog$ or hermeneutic$ or "field study" or fieldwork or "field work" or narrat$ or "lived experience$1" or "life experience$1" or "key informant$1" or "mixed method$" or "multi-method$" or "meta-synthesis" or survey$).ti,ab. (594625)

20 (("semi-structured" or semistructured or unstructured or informal or "in-depth" or indepth or "face-to-face" or structured or guide$ or "open end") adj3 (interview$1 or discussion$1 or questionnaire$1)).ti,ab. (70991)

21 16 or 17 or 18 or 19 or 20 (655524)

22 4 and 14 and 15 (2457)

23 limit 22 to "qualitative (best balance of sensitivity and specificity)" (580)

24 21 and 22 (159)

25 23 or 24 (677)
