# Supplementary material for: Clinicians’ views and experiences of prescribing oral anticoagulants for stroke prevention in atrial fibrillation: A qualitative meta-synthesis
Source: PLoS One. 2020 May 7;15(5):e0232484. doi: 10.1371/journal.pone.0232484 (PMC7205240; doi:10.1371/journal.pone.0232484)
Supplement: S1 File — (DOCX) [file pone.0232484.s003.docx]

**Supplementary Material**

**S1 Table: Reasons for exclusion of full text records**

| **Record** | **Year** | **Reason for exclusion** |
| --- | --- | --- |
| Aliot et al [1] | 2010 | Not qualitative methods |
| Alkhalil et al [2] | 2011 | Not qualitative methods |
| Alkhalil et al [3] | 2014 | Conference abstract; no full paper available |
| Alonso-Coello et al [4] | 2015 | Not qualitative methods |
| Alonso-Coello et al [5] | 2008 | Not qualitative methods |
| Anderson et al [6] | 2005 | Not qualitative methods |
| Andrade et al [7] | 2016 | Not qualitative methods |
| Andrade et al [8] | 2015 | Not qualitative methods |
| Anguita-Sanchez et al [9] | 2016 | Not qualitative methods |
| Arepally et al [10] | 2010 | Conference abstract; no full paper available |
| Asteggiano [11] | 2015 | Conference abstract; no full paper available |
| Bajorek et al [12] | 2006 | Not about prescription |
| Bajorek et al [13] | 2007 | Not about prescription |
| Banner et al [14] | 2014 | Conference abstract; no full paper available |
| Bell et al [15] | 2015 | Not qualitative methods |
| Borgundvaag and Ovens [16] | 2004 | Not qualitative methods |
| Brehaut et al [17] | 2007 | Not qualitative methods |
| Bungard et al [18] | 2003 | Not qualitative methods |
| Bush and Tayback [19] | 1998 | Not qualitative methods |
| Changying and Yihong [20] | 2014 | Conference abstract; no full paper available |
| Chataway et al [21] | 2016 | Not qualitative methods |
| Christian et al [22] | 2011 | Conference abstract; full paper included in review |
| Cimminiello et al [23] | 2018 | Not qualitative methods |
| Cloutier et al [24] | 2016 | Not qualitative methods |
| Crawford et al [25] | 1997 | Not qualitative methods |
| David et al [26] | 2013 | Not qualitative methods |
| Deplanque et al [27] | 2004 | Not qualitative methods |
| Devereaux et al [28] | 2001 | Not qualitative methods |
| Dharmarajan et al [29] | 2006 | Not qualitative methods |
| Dinh et al [30] | 2007 | Not qualitative methods |
| Douketis et al [31] | 2000 | Not qualitative methods |
| Eikelboom and Merli [32] | 2016 | Not qualitative methods |
| El Wakeel et al [33] | 2016 | Conference abstract; no full paper available |
| Farzin [34] | 2012 | Not qualitative methods |
| Ferguson et al [35] | 2016 | Not qualitative methods |
| Ferguson et al [36] | 2017 | Conference abstract; no full paper available |
| Flaker et al [37] | 2016 | Not qualitative methods |
| Frankel et al [38] | 2015 | Not qualitative methods |
| Fuchs et al [39] | 2015 | Not qualitative methods |
| Fujino et al [40] | 2014 | Conference abstract; no full paper available |
| Garavalia et al [41] | 2011 | Conference abstract; no full paper available |
| Gattellari et al [42] | 2008 | Not qualitative methods |
| Gattellari et al [43] | 2008 | Not qualitative methods |
| Gattellari et al [44] | 2015 | Conference abstract; no full paper available |
| Generalova et al [45] | 2019 | Not qualitative methods |
| Glauser et al [46] | 2016 | Not qualitative methods |
| Gross et al [47] | 2003 | Not qualitative methods |
| Heidbuchel et al [48] | 2018 | Not qualitative methods |
| Huang et al [49] | 2013 | Not qualitative methods |
| Holt et al [50] | 2018 | Not about prescription |
| Ingelgard et al [51] | 2006 | Not qualitative methods |
| Jia et al [52] | 2012 | Conference abstract; no full paper available |
| Kea et al [53] | 2017 | Conference abstract; no full paper available |
| Kirby et al [54] | 2018 | Not qualitative methods |
| Kirby et al [55] | 2016 | Conference abstract; no full paper available |
| Kristoffersen et al [56] | 2011 | Conference abstract; no full paper available |
| Kutner et al [57] | 1991 | Not qualitative methods |
| Leung et al [58] | 2016 | Conference abstract; no full paper available |
| Levitan et al [59] | 2013 | Conference abstract; no full paper available |
| Linchak et al [60] | 2014 | Not qualitative methods |
| Liu et al [61] | 2016 | Not qualitative methods |
| Maeda et al [62] | 2004 | Not qualitative methods |
| McCrory et al [63] | 1995 | Not qualitative methods |
| Mead et al [64] | 1996 | Not qualitative methods |
| Monette et al [65] | 1997 | Not qualitative methods |
| Moulson et al [66] | 2017 | Not qualitative methods |
| Muller-Oerlinghausen et al [67] | 2003 | Not qualitative methods |
| Nicholls et al [68] | 2014 | Not qualitative methods |
| Oehrlein et al [69] | 2017 | Conference abstract; no full paper available |
| Okamura et al [70] | 2015 | Not qualitative methods |
| Okamura et al [71] | 2012 | Conference abstract; no full paper available |
| Okumura et al [72] | 2012 | Conference abstract; no full paper available |
| Oqab et al [73] | 2014 | Not qualitative methods |
| Oqab et al [74] | 2015 | Not qualitative methods |
| Patzer et al [75] | 2016 | Not qualitative methods |
| Peterson et al [76] | 2002 | Not qualitative methods |
| Pokorney et al [77] | 2016 | Conference abstract; no full paper available |
| Pradhan and Levine [78] | 2002 | Not qualitative methods |
| Protasov [79] | 2013 | Not qualitative methods |
| Putnam et al [80] | 2004 | Not qualitative methods |
| Rada et al [81] | 2016 | Not qualitative methods |
| Raptis et al [82] | 2017 | Not qualitative methods |
| Salinas et al [83] | 2012 | Conference abstract only; no full paper available |
| Shen et al [84] | 2008 | Not qualitative methods |
| Sudlow et al [85] | 1998 | Not qualitative methods |
| Taylor et al [86] | 2015 | Not qualitative methods |
| Vasishta et al [87] | 2001 | Not qualitative methods |
| York et al [88] | 2003 | Not qualitative methods |

**S2 Table: Study quality**

|  | Are the results valid? | Is a qualitative methodology appropriate? | Was the research design appropriate to address the aims of the research? | Was the recruitment strategy appropriate to the aims of the research? | Was the data collected in a way that addressed the research issue? | Has the relationship between researcher and participants been adequately considered? | Have ethical issues been taken into consideration? | Was the data analysis sufficiently rigorous? | Is there a clear statement of findings? |
| --- | --- | --- | --- | --- | --- | --- | --- | --- | --- |
| Freeman et al. 2001 [89] | Yes | Yes | Yes | Can’t tell | Yes | Can’t tell | Yes | Can’t tell | Yes |
| Lipman et al.  2004 [90] | Yes | Yes | Yes | Yes | Yes | Yes | Can’t tell | Yes | Yes |
| Anderson et al. 2007 [91] | Yes | Yes | Yes | Can’t tell | Yes | No | Can’t tell | Can’t tell | Yes |
| Murray et al.  2011 [92] | Yes | Yes | Yes | Can’t tell | Yes | No | Can’t tell | Can’t tell | Can’t tell |
| Decker et al. 2012 [93] | Yes | Yes | Yes | Yes | Yes | No | Yes | Can’t tell | Yes |
| Bajorek et al.  2015 [94] | Yes | Yes | Yes | Yes | Yes | Can’t tell | Can’t tell | No | Yes |
| Borg Xuereb et al. 2016 [95] | Yes | Yes | Yes | Yes | Yes | No | Can’t tell | Can’t tell | Yes |
| Kirley et al. 2016 [96] | Yes | Yes | Yes | Can’t tell | Yes | Can’t tell | Can’t tell | Can’t tell | Yes |
| Wang et al. 2016 [97] | Yes | Yes | Yes | Yes | Yes | No | Can’t tell | Can’t tell | Yes |
| Karcher et al. 2016 [98] | Yes | Yes | Yes | Yes | Yes | No | Can’t tell | No | Can’t tell |
| Ferguson et al [99] | Yes | Yes | Yes | Can’t tell | Can’t tell | No | Can’t tell | Can’t tell | Can’t tell |
| Aarnio et al. 2019 [100] | Yes | Yes | Yes | Can’t tell | Can’t tell | No | No | Can’t tell | Yes |
| Kea et al. 2019 [101] | Yes | Yes | Yes | Yes | Yes | Can’t tell | Yes | Yes | Yes |

**S3 Table: Study characteristics**

| Study characteristics | Aim | Participants | Qualitative approach/ methods | Data analysis methods | Findings |
| --- | --- | --- | --- | --- | --- |
| Freeman et al. 2001, [89]  South West England, UK | To explore the reasons why GPs don’t always implement best practice. | Primary care  19 GPs  3-25 years’ experience as a partner. 14 members of the Royal College of General Practitioners, 7 GP trainers. | Three groups of GPs from separate geographical areas took part in multiple focus groups. Two of the groups met six times and one met once. GPs discussed the case notes of a patient encounter during which they did not follow evidence based medicine (EBM) and the feelings this generated. | Grounded theory approach to coding. | (Not all related to AF)Six themes influenced the implementation process: previous personal and professional experiences; the patient-doctor relationship; perceived tension between primary and secondary care; their feelings about their patients and the evidence; logistical problems. GPs were aware their choice of words influenced patients’ decisions. |
| Lipman et al.  2004, [90]  Northern England, UK | To explore how GP’s with an active interest in research or evidence based medicine make decisions about anticoagulation in AF patients. | Primary care  12 GPs (9/12 male, 5/12 full time, ages 33-55 years).  (1 poorly recorded interview discarded.)  Two were EBM workshop tutors; six others had formal EBM or critical appraisal training. Seven had academic appointments; 11 were research active. | Semi-structured interviews. Interview method took a constructionist approach, exploring the world that existed between GPs attempting to use research evidence in their practice and the investigator (a GP). Transcription in full then checked by the investigator. | Framework analysis. Reflexive discussions with researchers throughout the analysis. Coding after all interviews transcribed.  Used Potter & Wetherell’s criteria for validity: reflexive analysis through challenges to interpretation and presenting substantial extracts to the reader. | GP’s perception of the ‘evidence’ behind anticoagulant treatment was influenced by their individual experience, attitudes and knowledge of the literature. GPs supportive of EBM were committed to shared decision making, regardless of the treatment chosen by the patient. Hospital doctors were seen as being disease centred, difficult to challenge and poor at communicating. |
| Anderson et al. 2007 [91]  Leeds, UK | To improve understanding of physicians behaviour and attitudes in respect to decision making in AF and the use of anti-thrombotics. | Secondary and tertiary care  14 senior consultants or specialist registrars in Cardiology (5) or general medicine or geriatric medicine (9).  All were practicing clinicians regularly making OAC prescribing decision in AF, and worked at a large UK teaching hospital that undertakes both secondary and tertiary care. | Physicians considered vignettes and gave their opinion in treatment.  Semi structured interviews were conducted. | Analysis of interview transcripts used grounded theory.  Triangulation by use of open and closed questions and by reading emergent themes alongside AF and risk literature. | Marked variation in treatment recommendation. Greater likelihood of warfarin prescription to patients with previous intracerebral haemorrhage than to those with history of falls. Decision making in AF often involved uncertainty and concerns about knowledge of risk and benefit. |
| Murray et al.  2011, [92]  Rural, suburban and urban sites across Canada | 1. To determine attitudes & gaps in knowledge, skill and competence of community physicians in screening, diagnosis, treatment, management and referral.  2. To provide evidence for development of educational interventions. | Primary and secondary care  Clinicians:  28 HCPs interviewed:  family physicians/GPs; internal medicine specialists; cardiologists; emergency physicians and neurologists, all providing care to a minimum number of AF patients per month.  Patients: diagnosed for at least one year and had seen a physician ≥ twice in the last year. | Mixed method.  Quantitative survey recording clinicians knowledge, perceived barriers to optimal care, views on different AF prevention types, role in AF care and response to 3 case studies.  Qualitative open-ended semi-structures telephone interviews probing screening, diagnosis, treatment, management, referral and communication. | Interviews transcribed and analysed using NVIVO 7. Initial open coding was carried out based on the previously identified research questions and conceptual framework; followed by selective coding whereby data were coded and classified based on the continuum of care and Can MEDS competencies. | Barriers to optimal AF care included an unclear definition of AF, uncertainty of its pathology, knowledge gaps in screening, diagnosis and treatment. Specific challenges included clinical decision making, individualised patient therapy, communication with patients and between professionals and application of guidelines. |
| Decker et al. 2012, [93]  A large Midwestern city, USA | To define key issues in the prescription of warfarin therapy for AF by cardiology specialists and IM physicians | Secondary and tertiary care  27 HCPs: 18 cardiology physicians; 3 cardiology nurses; 5 internal medicine physicians; 1 internal medicine nurse practitioner.  Purposive sampling for those with experience of anticoagulation in AF. | In-depth interviews conducted in person or via telephone. A semi-structured interview guide was used. | Review of transcripts to develop familiarity; manual coding. (An iterative process) | In addition to CHADS2 score, patients’ social situation and past medication-taking behaviour were considered when treating them. Other themes: patient knowledge; real-world problems; breakdown in communication; clinical reluctance. |
| Bajorek et al.  2015, [94]  New South Wales, Australia | To describe Australian GPs approach to managing AF, including stroke prevention & identify range of services used to support patient care. | Primary care  50 GPs from ‘General practice’ or ‘Medical locals’ originally recruited to an intervention trial of stroke prevention in AF.  Average age: 53.75 +-9.94 years old; average 22.88+- 10.14 years in practice; 35/50 were male | Project officers clarified the nature of the services provided by each practice and conducted a structured questionnaire with both quantitative and qualitative responses, on paper, in person. | Qualitative: thematic analysis of open ended responses to specific questions, using manual inductive coding by the project officer verified by the lead researcher. | Key determinants influencing GPs’ initiation of OACs were: stroke risk/CHADS₂ score followed by patients’ adherence/compliance. GPs focused more on medication safety and day-to-day management of therapy than on risk of bleeding. |
| Borg Xuereb et al. 2016, [95]  West Midlands, UK | To explore patients’ and physicians’ experiences of AF consultations and OAC decision-making | Primary and secondary care  Five subgroups of participants:  16 HCPs: 4 consultant cardiologist; 4 consultant general physicians; 4 general practitioners; 4 cardiology registrars and 11 AF patients.  Physicians were eligible for inclusion if they had experience of managing patients with AF and had prescribed warfarin. | Semi-structured interviews | Multi-perspective interpretive phenomenological analysis | Themes representing patients’ experiences: Positioning within the physician-patient dyad; health-life balance; drug myths and fear of stroke.  Themes representing physicians’ experiences: Mechanised metaphors and probabilities; navigating towards the ‘right’ decision; negotiating systemic factors. |
| Kirley et al. 2016  [96]  Chicago, USA | To address the many unanswered questions about physician knowledge, attitudes, and practices regarding NOAC use. | Primary, secondary and tertiary care.  7 HCPs: 3 family physicians; 1 internist; 2 cardiologists; 1 cardiologist specialising in electrophysiology. | Semi-structured open-ended interviews | Familiarisation and independent coding by 2 researchers. Thematic analysis, data then indexed into a framework matrix. | 1. The likelihood of physicians to prescribe NOACs depends upon their willingness to try new medications and their successful experience with them. 2. Physicians balance the benefits and risks of anticoagulation in AF patients, although not always accurately. 3. Patient and physician convenience and preferences are important. The out-of-pocket cost of NOACs deter prescription. |
| Wang et al. 2016, [97]  Sydney, Australia | To describe HCP perspectives on the decision-making around antithrombothic therapy for stroke prevention in AF | Primary and secondary care  26 HCPs: Seven pharmacists; seven specialist clinicians; 6 GPs and six nurses.  Nurses and pharmacists were from primary and secondary care. Only health professionals with experience in prescribing and managing antithrombotics for stroke prevention in AF were recruited. | Semi-structured face-to-face interviews with open-ended questions were conducted until theme saturation was reached in each HCP subgroup.  Transcripts were transcribed verbatim. | Manual inductive coding was conducted by 2 researchers independently, using Thematic analysis techniques. The analysis was validated by 3 further independent researchers. | 1. HCPs focused on stroke risk not bleeding risk or medication safety issues – a more comprehensive assessment is needed.  2. Different HCP types had different antithrombotic preferences (warfarin/NOAC).  3. Different HCPs had different priorities: GPs and specialists focused on the appropriate prescription of antithrombotics; pharmacists and nurses focused on daily management. |
| Karcher et al. 2016, [98]  11 states in South Eastern USA termed the ‘Stroke Belt’ | To identify current practice patterns and barriers (including racial disparities) to optimal stroke prevention in AF patients in the US ‘Stroke Belt’ | Primary and secondary care  14 HCPs interviewed: 7 physicians, 5 nurses, 2 medical assistants.  Clinicians were from 3 cardiologist and 3 primary care practices.  Practices included if they provided care to ≥20 patients for cardiologists or ≥10 for PCPs; had ≥20% minority patients & ≥10% patients of low socioeconomic status  All physicians were board certified and averaged 25 years in clinical practice. | Mixed methods: web-based survey; site visits to discern baseline practice behaviours and areas for improvement; one-on-one interviews using standardised questionnaires including props to facilitate expansion if needed | Qualitative analysis: transcripts and researcher notes from interviews extensively reviewed to identify overarching themes that were categorised into topics | Identified contributors to racial disparities in stroke prevention included implicit racial biases, lack of awareness of racial disparities in stroke risk, lack of multicultural awareness and training. General barriers included lack of lack of stroke assessment consistency in assessing stroke and bleeding risk; underuse of standardised risk assessment tools; discomfort with NOACS and patient education deficiencies. |
| Ferguson et al. [99]  2017,  Sydney, Australia | To elucidate patient and provider barriers and enablers to adherence to OACs in patients with chronic heart failure & | Tertiary care  Clinician’s notes from the treatment of 144 patients with Chronic heart failure and concomitant AF enrolled in an over-arching cohort study April-October 2013. Patients exclusions: <18years old, AF due to irreversible causes such as thyrotoxicosis | Patient interviews during bed-side clinical assessments and review of healthcare file notes. | Synthesis of patient interviews & clinician notes. Data analysed within the WHO’s multidimensional model of medication adherence (socio-economic, health system, condition, treatment & patient barriers to anticoagulation | Clinician related findings: barriers to effective anti-coagulation included patients experiencing psychiatric illness, cognitive impairment and depression; clinician reticence due to fear of falls, frailty, age, bleeding and the challenges of multi-morbidity. |
| Aarnio et al.  [100]  2019,  Central and Eastern Finland | To understand  how physicians choose an oral anticoagulant (OAC) for patients with AF and  how physicians view patients’ participation in this decision | Primary and secondary care  17 HCPs: 8 GPs; 2 neurologists; 5 cardiologists; 2 internal medicine specialists.  14 male; 3 female.  Frequency of treating AF patients ranged from <monthly to daily. | Semi-structured interviews with open-ended questions. 13 face-to-face and 4 phone interviews. Interviews conducted by 1 researcher; recorded and transcribed verbatim by a transcription service. | Thematic analysis using inductive and deductive approaches.  Deductive: previously known factors looked for in data.  Inductive: data searched for new factors. | When there were no contraindications  or other clinical factors limiting the choice between OACs, patient's opinion was the most influential factor in the prescription decision.  New influential factors: indication (the need for cardioversion), physicians’ past OAC prescribing habits, characteristics of clinical trials and patients’ entitlement to reimbursement affecting the amount of co‐payment. |
| Kea et al  [101]  2019,  Oregon, USA | To examine emergency physician thought  processes and identify themes that prevent or support oral  anticoagulation prescribing for new-onset atrial fibrillation | Secondary care  18 HCPs: Board-prepared or board-certified Emergency department attending physicians who had evaluated a new onset AF patients in the last 30 days | Semi-structured telephone interviews conducted by a resident physician skilled in conducting interviews. Interview guide revised based on participants’ answers. | A modified, constructivist  grounded theory approach to both data collection and analysis.  Comparative coding by 3 researchers, followed by thematic analysis. | Three broad themes are reported relating to OAC prescription in the emergency department: 1. OAC prescribing practice including clinician practice patterns, beliefs and barriers and patient factors including co-morbidities, bleeding risk, social concerns. 2. Lack of guideline usage. 3. The need for population specific guidelines that include patient social factors. |

**S4 Table: Frequency of codes in studies**

|  | Code | | | | | | | | | | | | |
| --- | --- | --- | --- | --- | --- | --- | --- | --- | --- | --- | --- | --- | --- |
| Study | Clinicians' treatment or risk assessment knowledge and understanding | Clinicians' sense of uncertainty, anxiety, guilt and responsibility | Clinicians' treatment preferences and their reasons | Clinicians' treatment safety concerns | Clinicians' use of scientific evidence and its value | Clinicians' communication with patients | Clinicians' use of persuasion | Clinicians' view of who is the final decision maker | Clinicians' outlook (disease vs patient centred) | Clinicians' perception of the medical hierarchy | Communication between HCPs and continuity of care | Patients' social and clinical factors | Patients' treatment preferences and experiences |
| Freeman et al. 2001 [89] | 0 | 2 | 0 | 0 | 1 | 1 | 1 | 0 | 0 | 0 | 1 | 0 | 0 |
| Lipman et al. 2004 [90] | 3 | 3 | 3 | 2 | 9 | 2 | 1 | 4 | 2 | 2 | 4 | 0 | 2 |
| Anderson et al. 2007 [91] | 4 | 4 | 4 | 1 | 0 | 2 | 0 | 4 | 0 | 0 | 0 | 2 | 0 |
| Murray et al. 2011 [92] | 1 | 1 | 1 | 0 | 0 | 1 | 0 | 0 | 0 | 0 | 0 | 0 | 0 |
| Decker et al. 2012 [93] | 2 | 3 | 1 | 2 | 1 | 0 | 0 | 0 | 0 | 0 | 0 | 3 | 2 |
| Bajorek et al. 2015 [94] | 3 | 0 | 0 | 1 | 0 | 0 | 0 | 0 | 0 | 1 | 0 | 2 | 0 |
| Borg-Xuereb et al. 2016 [95] | 0 | 0 | 0 | 0 | 2 | 7 | 1 | 2 | 0 | 0 | 5 | 2 | 1 |
| Kirley et al. 2016 [96] | 1 | 0 | 9 | 3 | 2 | 0 | 0 | 1 | 0 | 1 | 0 | 7 | 3 |
| Wang et al. 2016 [97] | 9 | 7 | 7 | 12 | 3 | 1 | 2 | 1 | 4 | 5 | 1 | 9 | 5 |
| Karcher et al. 2016 [98] | 3 | 1 | 2 | 0 | 0 | 1 | 0 | 0 | 0 | 0 | 0 | 5 | 0 |
| Ferguson et al. 2017 [99] | 1 | 0 | 2 | 0 | 0 | 0 | 0 | 0 | 0 | 0 | 0 | 2 | 0 |
| Aarnio et al. 2019 [100] | 0 | 0 | 0 | 0 | 1 | 0 | 0 | 2 | 0 | 0 | 0 | 2 | 4 |
| Kea et al. 2019 [101] | 11 | 0 | 5 | 1 | 4 | 0 | 0 | 1 | 1 | 5 | 5 | 15 | 1 |

**References**

1. Aliot E, Breithardt G, Brugada J, Camm J, Lip GYH, Vardas PE, et al. An international survey of physician and patient understanding, perception, and attitudes to atrial fibrillation and its contribution to cardiovascular disease morbidity and mortality. Europace. 2010;12(5):626-33.

2. Alkhalil M, Quinn S, Magill P, Tauro R, Prabhavalkar S. Atrial fibrillation in paced rhythm: ''under- diagnosed and under-recognized''. Irish Journal of Medical Science. 2011;180:S406-S7.

3. Alkhalil M, Cromie N. Education in atrial fibrillation. Irish Journal of Medical Science. 2014;183(8 SUPPL. 1):S419.

4. Alonso-Coello P, Montori VM, Diaz MG, Devereaux PJ, Mas G, Diez AI, et al. Values and preferences for oral antithrombotic therapy in patients with atrial fibrillation: physician and patient perspectives. Health expectations : an international journal of public participation in health care and health policy. 2015;18(6):2318-27.

5. Alonso-Coello P, Montori VM, Sola I, Schunemann HJ, Devereaux P, Charles C, et al. Values and preferences in oral anticoagulation in patients with atrial fibrillation, physicians' and patients' perspectives: protocol for a two-phase study. BMC health services research. 2008;8:221.

6. Anderson DR, Gardner MJ, Putnam W, Jassal D, Brownell B, Flowerdew G, et al. Population-based evaluation of the management of antithrombotic therapy for atrial fibrillation. The Canadian journal of cardiology. 2005;21(3):257-66.

7. Andrade JG, Krahn AD, Skanes AC, Purdham D, Ciaccia A, Connors S. Values and Preferences of Physicians and Patients With Nonvalvular Atrial Fibrillation Who Receive Oral Anticoagulation Therapy for Stroke Prevention. The Canadian journal of cardiology. 2016;32(6):747-53.

8. Andrade J, Ciaccia A, Krahn AD, Purdham D, Skanes A, Connors S. Attitudes, values and preferences of physicians and patients with non-valvular atrial fibrillation receiving oral anticoagulation therapy for stroke prevention. Canadian Journal of Cardiology. 2015;31(10 SUPPL. 1):S303-S4.

9. Anguita-Sanchez M, Marco-Vera P, Alonso-Moreno FJ, Arribas-Ynsaurriaga F, Gallego-Cullere J, Honorato-Perez J, et al. Perception of physicians on factors that influence the choice of a dicoumarin or a new oral anticoagulant in patients with non-valvular atrial fibrillation. Aten Primaria. 2016;48(8):527-34.

10. Arepally G, Bauer KA, Bhatt DL, Merli GJ, Naccarelli GV, Carter RD, et al. The use of antithrombotic therapies in the prevention and treatment of arterial and venous thrombosis: a survey of current knowledge and practice supporting the need for clinical education. Critical pathways in cardiology. 2010;9(1):41-8.

11. Asteggiano R, Guenoun M, Baron Esquivias G, Richter D. CCP survey about management of thromboembolic events in atrial fibrillation prevention. European Heart Journal. 2015;36:1078.

12. Bajorek BV, Krass I, Ogle SJ, Duguid MJ, Shenfield GM. Warfarin use in the elderly: The nurses' perspective. Australian Journal of Advanced Nursing. 2006;23(3):19-25.

13. Bajorek BV, Ogle SJ, Duguid MJ, Shenfield GM, Krass I. Management of warfarin in atrial fibrillation: views of health professionals, older patients and their carers. The Medical journal of Australia. 2007;186(4):175-80.

14. Banner DJ, King-Shier K, Janke F, Clark A, Hadi H, MacLeod M, et al. Atrial fibrillation in rural and Northern Canada: A qualitative study of primary care providers managing atrial fibrillation and enacting clinical guidelines. Canadian Journal of Cardiology. 2014;30(10 SUPPL. 1):S293.

15. Bell S, Randall M, Al-Shahi Salman R, Abousleiman Y, Ahmad N, Ahmed A, et al. Oral anticoagulation after intracranial haemorrhage: A survey of UK stroke physicians. International Journal of Stroke. 2015;10:28-9.

16. Borgundvaag B, Ovens H. Cardioversion of uncomplicated paroxysmal atrial fibrillation: a survey of practice by Canadian emergency physicians. CJEM: Canadian Journal of Emergency Medicine. 2004;6(3):155-60 6p.

17. Brehaut JC, Poses R, Shojania KG, Lott A, Man-Son-Hing M, Bassin E, et al. Do physician outcome judgments and judgment biases contribute to inappropriate use of treatments? Study protocol. Implementation science : IS. 2007;2:18.

18. Bungard TJ, Ghali WA, McAlister FA, Buchan AM, Cave AJ, Hamilton PG, et al. The relative importance of barriers to the prescription of warfarin for nonvalvular atrial fibrillation. The Canadian journal of cardiology. 2003;19(3):280-4.

19. Bush D, Tayback M. Anticoagulation for nonvalvular atrial fibrillation: effects of type of practice on physicians' self-reported behavior. The American journal of medicine. 1998;104(2):148-51.

20. Changying W, Yihong S. A cross-sectional survey on the perception of the anticoagulant treatment in atrial fibrillation in physicians from county Hospitals. Journal of the American College of Cardiology. 2014;64(16 SUPPL. 1):C244.

21. Chataway J, Lichten C, Clark E. The future of anticoagulation. Future cardiology. 2016;12(1):5-7.

22. Christian BX, Rachel LS, Danielle ES, Gregory YHL, Deirdre AL. Patients' and physicians' experiences of atrial fibrillation and anticoagulant therapy: A qualitative journey. Psychology & Health. 2011;26:255-6.

23. Cimminiello C, Hatala R, Pakarinen S, Polo Friz H, Fitzmaurice D, Hobbs FR. Non-vitamin k antagonist oral anticoagulants in a European primary care physician survey. BJGP open. 2018;2(3):bjgpopen18X101602.

24. Cloutier J, Khoo C, Hiebert B, Wassef A, Seifer C. Physician decision making in anticoagulating atrial fibrillation: A prospective survey evaluating a physician alert system for atrial fibrillation detected on cardiac implantable electronic devices. Journal of the American College of Cardiology. 2016;67(13 SUPPL. 1):837.

25. Crawford PJ, Hendry A, Mallinson A, MacDonald JB. Anticoagulant services in West Glasgow: accessibility for elderly patients. Health Bulletin. 1997;55(6):394-8 5p.

26. David C, Fedrizzi S, Le Bellec ML, Lacotte J, Mosquet B, Coquerel A. Situation of the prescriber's knowledge about the new anticoagulants dabigatran and rivaroxaban. Fundamental and Clinical Pharmacology. 2013;27:59.

27. Deplanque D, Leys D, Parnetti L, Schmidt R, Ferro J, De Reuck J, et al. Stroke prevention and atrial fibrillation: reasons leading to an inappropriate management. Main results of the SAFE II study. British journal of clinical pharmacology. 2004;57(6):798-806.

28. Devereaux PJ, Anderson DR, Gardner MJ, Putnam W, Flowerdew GJ, Brownell BF, et al. Differences between perspectives of physicians and patients on anticoagulation in patients with atrial fibrillation: observational study. BMJ (Clinical research ed). 2001;323(7323):1218-22.

29. Dharmarajan TS, Varma S, Akkaladevi S, Lebelt AS, Norkus EP. To anticoagulate or not to anticoagulate? A common dilemma for the provider: physicians' opinion poll based on a case study of an older long-term care facility resident with dementia and atrial fibrillation. Journal of the American Medical Directors Association. 2006;7(1):23-8.

30. Dinh T, Nieuwlaat R, Tieleman RG, Buller HR, van Charante NAM, Prins MH, et al. Antithrombotic drug prescription in atrial fibrillation and its rationale among general practitioners, internists and cardiologists in The Netherlands--The EXAMINE-AF study. A questionnaire survey. International journal of clinical practice. 2007;61(1):24-31.

31. Douketis JD, Crowther MA, Cherian SS. Perioperative anticoagulation in patients with chronic atrial fibrillation who are undergoing elective surgery: results of a physician survey. The Canadian journal of cardiology. 2000;16(3):326-30.

32. Eikelboom J, Merli G. Bleeding with direct oral anticoagulants vs warfarin: clinical experience. The American journal of emergency medicine. 2016;34(11S):3-8.

33. El Wakeel LM, Sabry S, Saleh MA. Clinical pharmacist-managed anticoagulation service in atrial fibrillation patients: an Egyptian experience. Pharmacotherapy. 2016;36(7):E86-E7.

34. Farzin A. Status and challenges of oral anticoagulant treatment, in atrial fibrillation in general practice. Survey among 133 general practitioners in Picardy. Etat des lieux et difficultés de la prise en charge par antivitamine K dans la fibrillation auriculaire en médecine générale (enquête auprès de 133 médecins généralistes picards). [U - Thesis ; (Thesis)]. In press 2012.

35. Ferguson C, Inglis SC, Newton PJ, Middleton S, Macdonald PS, Davidson PM. Education and practice gaps on atrial fibrillation and anticoagulation: a survey of cardiovascular nurses. BMC medical education. 2016;16:9.

36. Ferguson C, Hickman L, Phillips J, Newton P, Inglis S, Lam L, et al. Exploring the acute care registered nurses' contribution in anticoagulation decision making for stroke prevention in atrial fibrillation. Int J Stroke. 2017;12(3 Supplement 1):48.

37. Flaker GC, Theriot P, Binder LG, Dobesh PP, Cuker A, Doherty JU. Management of Periprocedural Anticoagulation: A Survey of Contemporary Practice. Journal of the American College of Cardiology. 2016;68(2):217-26.

38. Frankel DS, Parker SE, Rosenfeld LE, Gorelick PB. HRS/NSA 2014 survey of atrial fibrillation and stroke: Gaps in knowledge and perspective, opportunities for improvement. Heart rhythm : the official journal of the Heart Rhythm Society. 2015;12(8):e105-13.

39. Fuchs P, Vogel T, Lang PO. Anticoagulation in the aged patient with atrial fibrillation: What are prescribing cardiologists, geriatricians and general practitioners? Revue de Medecine Interne. 2015;36(8):509-15.

40. Fujino T, Kobayashi K, Kinoshita T, Yuzawa H, Suzuki T, Abe A, et al. Survey of general practitioners on the actual conditions of atrial fibrillation treatment, including anticoagulation therapy. European Heart Journal. 2014;35:1103.

41. Garavalia LS, Garavalia BJ, Simon T, Daniel W, Decker C. Weighing more than the risks: Cardiologists' prescription of warfarin for atrial fibrillation patients. Circulation: Cardiovascular Quality and Outcomes. 2011;4(6 MeetingAbstracts2011):no pagination.

42. Gattellari M, Worthington J, Zwar N, Middleton S. Barriers to the use of anticoagulation for nonvalvular atrial fibrillation - A representative survey of Australian family physicians. Stroke. 2008;39(1):227-30.

43. Gattellari M, Worthington JM, Zwar NA, Middleton S. The management of non-valvular atrial fibrillation (NVAF) in Australian general practice: bridging the evidence-practice gap. A national, representative postal survey. BMC Fam Pract. 2008;9:62.

44. Gattellari M, Worthington JM, Leung DY. Novel anticoagulants for stroke prevention in atrial fibrillation: A survey of Australian GPs. International Journal of Stroke. 2015;10:62.

45. Generalova D, Cunningham S, Leslie SJ, Rushworth G, McIver L, Stewart D. Prescribers' views and experiences of using direct acting oral anticoagulants in the management of nonvalvular atrial fibrillation: A survey in remote and rural Scotland. Br J Clin Pharmacol. 2019;85(10):2414-22.

46. Glauser TA, Barnes J, Nevins H, Cerenzia W. The Educational Needs of Clinicians Regarding Anticoagulation Therapy for Prevention of Thromboembolism and Stroke in Patients With Atrial Fibrillation. American journal of medical quality : the official journal of the American College of Medical Quality. 2016;31(1):38-46.

47. Gross CP, Vogel EW, Dhond AJ, Marple CB, Edwards RA, Hauch O, et al. Factors influencing physicians' reported use of anticoagulation therapy in nonvalvular atrial fibrillation: a cross-sectional survey. Clinical therapeutics. 2003;25(6):1750-64.

48. Heidbuchel H, Dagres N, Antz M, Kuck KH, Lazure P, Murray S, et al. Major knowledge gaps and system barriers to guideline implementation among European physicians treating patients with atrial fibrillation: a European Society of Cardiology international educational needs assessment. Europace. 2018;20(12):1919-28.

49. Huang C, Siu M, Vu L, Wong S, Shin J. Factors influencing doctors' selection of dabigatran in non-valvular atrial fibrillation. Journal of evaluation in clinical practice. 2013;19(5):938-43.

50. Holt TA, Dalton AR, Kirkpatrick S, Hislop J, Marshall T, Fay M, et al. Barriers to a software reminder system for risk assessment of stroke in atrial fibrillation: a process evaluation of a cluster randomised trial in general practice. Br J Gen Pract. 2018;68(677):e844-e51.

51. Ingelgard A, Hollowell J, Reddy P, Gold K, Tran K, Fitzmaurice D. What are the barriers to warfarin use in atrial fibrillation?: Development of a questionnaire. J Thromb Thrombolysis. 2006;21(3):257-65.

52. Jia J. Analysis of physicians awareness level to preventive stroke in China. Cerebrovasc Dis. 2012;34:63.

53. Kea B, Robinson C, Zhu A, Livingston J, Sun B. Barriers to prescribing stroke prophylaxis for atrial fibrillation in the emergency department: A qualitative provider perspective. Ann Emerg Med. 2017;70(4 Supplement 1):S119.

54. Kirby A, Murphy A, Bradley C. Multi-disciplinary decision making in general practice. Journal of Health Organization and Management. 2018;32(2):146-56.

55. Kirby A, Murphy A, Bradley C. Analysing the prescribing landscape for oral anticoagulants. Value in Health. 2016;19(7):A663.

56. Kristoffersen AH, Thue G, Ajzner E, Claes N, Aytekin M, Horvath AR, et al. How do clinicians monitor treatment with vitamin K antagonists: An international survey. Journal of Thrombosis and Haemostasis. 2011;9:446-7.

57. Kutner M, Nixon G, Silverstone F. Physicians' attitudes toward oral anticoagulants and antiplatelet agents for stroke prevention in elderly patients with atrial fibrillation. Archives of internal medicine. 1991;151(10):1950-3.

58. Leung L, McAllister M, Selim M, Fisher M. Factors influencing oral anticoagulant prescribing practices for atrial fibrillation among cardiologists, internists, and vascular neurologists. Neurology. 2016;86(16 SUPPL. 1):no pagination.

59. Levitan B, Yuan Z, Gonzalez JM, Hauber AB, Lees M, Piccini JP, et al. Patient and physician preferences in the united states for benefits and risks of anticoagulant use in atrial fibrillation-results from a conjoint-analysis study. Value in Health. 2013;16(3):A11.

60. Linchak RM, Kompaniets OG, Nedbaikin AM, Komkov DS, Iusova IA. [What physicians think and know about antithrombotic therapy in atrial fibrillation]. Kardiologiia. 2014;54(10):32-8.

61. Liu X, Baumgarten M, Smith G, Gambert S, Gottlieb S, Rattinger G, et al. Warfarin usage among elderly atrial fibrillation patients with traumatic injury, an analysis of United States Medicare fee-for-service enrollees. J Clin Pharmacol. 2015;55(1):25-32.

62. Maeda K, Sakai T, Hira K, Sato TS, Bito S, Asai A, et al. Physicians' attitudes toward anticoagulant therapy in patients with chronic atrial fibrillation. Internal medicine (Tokyo, Japan). 2004;43(7):553-60.

63. McCrory DC, Matchar DB, Samsa G, Sanders LL, Pritchett EL. Physician attitudes about anticoagulation for nonvalvular atrial fibrillation in the elderly. Archives of internal medicine. 1995;155(3):277-81.

64. Mead GE, Murray H, McCollum CN, O'Neill PA. How do general practitioners manage patients at risk from stroke? The British journal of clinical practice. 1996;50(8):426-30.

65. Monette J, Gurwitz JH, Rochon PA, Avorn J. Physician attitudes concerning warfarin for stroke prevention in atrial fibrillation: results of a survey of long-term care practitioners. Journal of the American Geriatrics Society. 1997;45(9):1060-5.

66. Moulson N, McIntyre WF, Oqab Z, Yazdan-Ashoori P, Quinn KL, van Oosten E, et al. The anticoagulation choices of internal medicine residents for stroke prevention in non-valvular atrial fibrillation. Postgrad Med J. 2017;93(1100):308-12.

67. Muller-Oerlinghausen B, Rostock T. Pharmacologic management of atrial fibrillation by specialists in internal medicine, cardiologists, and general practitioners (GPs) in Berlin. Med Klin. 2003;98(7):359-63.

68. Nicholls SG, Brehaut JC, Arim RG, Carroll K, Perez R, Shojania KG, et al. Impact of stated barriers on proposed warfarin prescription for atrial fibrillation: a survey of Canadian physicians. Thrombosis journal. 2014;12:13.

69. Oehrlein EM, Albrecht J, Perfetto EM, McCall D, Cooke CE, Slejko JF, et al. Listening to the "patient voice" to improve design and interpretation of secondary analyses: An example in atrial fibrillation. Value in Health. 2017;20(9):A773.

70. Okumura K, Inoue H, Yasaka M, Gonzalez JM, Hauber AB, Levitan B, et al. Comparing Patient and Physician Risk Tolerance for Bleeding Events Associated with Anticoagulants in Atrial Fibrillation-evidence from the United States and Japan. Value in Health Regional Issues. 2015;6:65-72.

71. Okumura K, Inoue H, Yasaka M, Gonzalez JM, Hauber AB, Iwamoto K, et al. Characteristics influencing japanese atrial fibrillation patients preferences for anticoagulants use. Value Health. 2012;15(7):A380-A1.

72. Okumura K, Inoue H, Yasaka M, Gonzalez JM, Hauber AB, Iwamoto K, et al. Japanese patients and physicians preferences for anticoagulants use in atrial fibrillation - Results from a conjoint-analysis study. Value Health. 2012;15(7):A380.

73. Oqab Z, McIntyre WF, Quinn K, Quadros K, Yazdan-Ashoori P, Van Oosten E, et al. Resident physician attitudes toward anticoagulation for stroke prevention in patients with nonvalvular atrial fibrillation. Can J Cardiol. 2014;30(10 SUPPL. 1):S311.

74. Oqab Z, McIntyre WF, Quinn KL, Lamb T, Quadros K, Yasdan-Ashoori P, et al. Update on a national survey on resident physicians attitudes towards anticoagulation for stroke prevention in atrial fibrillation. Can J Cardiol. 2015;31(10 SUPPL. 1):S263-S4.

75. Patzer J, Oqab Z, McIntyre WF, Hopman WM, Baranchuk A. Cardiology residents' anticoagulation preferences for stroke prophylaxis in atrial fibrillation patients. Int J Cardiol. 2016;223:63-4.

76. Peterson GM, Boom K, Jackson SL, Vial JH. Doctors' beliefs on the use of antithrombotic therapy in atrial fibrillation: identifying barriers to stroke prevention. Intern Med J. 2002;32(1-2):15-23.

77. Pokorney SD, Granger CB, Bloom D, Thomas KL, Al-Khatib SM, Anderson J, et al. Provider rather than patient misperceptions appear to be the most common barriers to oral anticoagulation use for stroke prevention in atrial fibrillation. Circulation. 2016;134(Supplement 1).

78. Pradhan AA, Levine MAH. Warfarin use in atrial fibrillation: A random sample survey of family physician beliefs and preferences. The Canadian journal of clinical pharmacology = Journal canadien de pharmacologie clinique. 2002;9(4):199-202.

79. Protasov K, Fedorishina O. Realities and Perspectives of Stroke Prevention in Atrial Fibrillation: Physician's Opinions Analysis. Kardiologiya. 2013;53(10):30-+.

80. Putnam W, Nicol K, Anderson D, Brownell B, Chiasson M, Burge FI, et al. Anticoagulation in atrial fibrillation. Is there a gap in care for ambulatory patients? Canadian family physician Medecin de famille canadien. 2004;50:1244-50.

81. Rada MC, Mandra M, Tomas F, Rollan I, Floridia J, Herrera G. Daily practice anticoagulant prescription amongst neurologist from Salta city. Neurologia Argentina. 2016;8(2):101-4.

82. Raptis S, Chen JN, Saposnik F, Pelyavskyy R, Liuni A, Saposnik G. Aversion to ambiguity and willingness to take risks affect therapeutic decisions in managing atrial fibrillation for stroke prevention: Results of a pilot study in family physicians. Patient Preference and Adherence. 2017;11:1533-9.

83. Salinas GD, Robinson CO, Roepke N, Burton BS, Susalka D, Cline K. Current attitudes and practice patterns of using new and emerging therapies to manage patients with atrial fibrillation (AF): A national assessment of cardiologists and primary care physicians. Circ Cardiovasc Qual Outcomes. 2012;5(3 SUPPL. 1):no pagination.

84. Shen Q, Cordato D, Ng J, Hung WT, Kokkinos J, Karr M, et al. Anticoagulant usage for primary stroke prevention: a general practitioner survey in local areas of metropolitan Sydney. J Clin Neurosci. 2008;15(2):166-71.

85. Sudlow M, Thomson R, Rodgers H, Livingstone S, Kenny RA. The effect of age and quality of life on doctors' decisions to anticoagulate patients with atrial fibrillation. Age Ageing. 1998;27(3):285-9.

86. Taylor J. Survey shows nearly 40% cardiologists do not follow atrial fibrillation guidelines. Eur Heart J. 2015;36(33):2200-1.

87. Vasishta S, Toor F, Johansen A, Hasan M. Stroke prevention in atrial fibrillation: physicians' attitudes to anticoagulation in older people. Arch Gerontol Geriatr. 2001;33(3):219-26.

88. York M, Agarwal A, Ezekowitz M. Physicians' attitudes and the use of oral anticoagulants: surveying the present and envisioning future. J Thromb Thrombolysis. 2003;16(1-2):33-7.

89. Freeman AC, Sweeney K. Why general practitioners do not implement evidence: qualitative study. BMJ. 2001;323(7321):1100-2.

90. Lipman T, Murtagh MJ, Thomson R. How research-conscious GPs make decisions about anticoagulation in patients with atrial fibrillation: a qualitative study. Fam Pract. 2004;21(3):290-8.

91. Anderson N, Fuller R, Dudley N. 'Rules of thumb' or reflective practice? Understanding senior physicians' decision-making about anti-thrombotic usage in atrial fibrillation. QJM. 2007;100(5):263-9.

92. Murray S, Lazure P, Pullen C, Maltais P, Dorian P. Atrial fibrillation care: challenges in clinical practice and educational needs assessment. Can J Cardiol. 2011;27(1):98-104.

93. Decker C, Garavalia L, Garavalia B, Simon T, Loeb M, Spertus JA, et al. Exploring barriers to optimal anticoagulation for atrial fibrillation: interviews with clinicians. J Multidiscip Healthc. 2012;5:129-35.

94. Bajorek B, Magin P, Hilmer S, Krass I. Contemporary approaches to managing atrial fibrillation: A survey of Australian general practitioners. Australas Med J. 2015;8(11):357-67.

95. Borg Xuereb C, Shaw RL, Lane DA. Patients' and physicians' experiences of atrial fibrillation consultations and anticoagulation decision-making: A multi-perspective IPA design. Psychol Health. 2016;31(4):436-55.

96. Kirley K, GouthamRao, Bauer V, Masi C. The Role Of NOACs in Atrial Fibrillation Management: A Qualitative Study. Journal of atrial fibrillation. 2016;9(1):1416.

97. Wang Y, Bajorek B. Decision-making around antithrombotics for stroke prevention in atrial fibrillation: the health professionals' views. Int J Clin Pharm. 2016;38(4):985-95.

98. Karcher R, Berman AE, Gross H, Hess DC, Jauch EC, Viser PE, et al. Addressing disparities in stroke prevention for atrial fibrillation: Educational opportunities. Am J Med Qual. 2016;31(4):337-48.

99. Ferguson C, Inglis SC, Newton PJ, Middleton S, Macdonald PS, Davidson PM. Barriers and enablers to adherence to anticoagulation in heart failure with atrial fibrillation: patient and provider perspectives. J Clin Nurs. 2017;26(23-24):4325-34.

100. Aarnio E, Huupponen R, Hameen-Anttila K, Merikoski M, Puhakka J, Korhonen MJ. Physicians' views on patient participation in choice of oral anticoagulants in atrial fibrillation-a qualitative study. Basic Clin Pharmacol Toxicol. 2019;124(4):416-22.

101. Kea B, Alligood T, Robinson C, Livingston J, Sun BC. Stroke Prophylaxis for Atrial Fibrillation? To Prescribe or Not to Prescribe-A Qualitative Study on the Decisionmaking Process of Emergency Department Providers. Ann Emerg Med. 2019;74(6):759-71.
